# Acute flaccid paralysis surveillance performance from 2011 to 2020 in Jonglei State, South Sudan: progress and challenges encountered

**DOI:** 10.11604/pamj.supp.2022.42.1.33966

**Published:** 2022-06-11

**Authors:** Jok Mayom Jil, Ayesheshem Ademe Tegegne, Sylvester Maleghemi, Kibebu Kinfu Berta, Tadesse Gossaye Birru, Ochan Taban David Kilo

**Affiliations:** 1 World Health Organization, South Sudan Country Office, Juba, South Sudan

**Keywords:** Acute flaccid paralysis surveillance, South Sudan, poliovirus, sensitivity, disease surveillance

## Abstract

**Introduction:**

South Sudan reported the last indigenous wild poliovirus (WPV) in 2001 in Unity State, while the country was part of Sudan. In addition, the country reported an imported case of WPV in 2004-2005 and 2008-2009. The WPV circulation in the state was interrupted in 2009 and the last case was reported in Ayod county. The country continues to be at risk of importation of circulating vaccine-derived poliovirus type 2 (cVDPV2). In 2014 and 2020 the country experienced an outbreak of cVDPV2, in which Jonglei state was one of the affected states. Four out of 50 (8%) cVDPV2 cases in 2020 were reported from Jonglei State. The purpose of this study is to review surveillance performance indicators of Jonglei and compare them with the WHO surveillance performance standard and other country´s surveillance performances.

**Methods:**

retrospective secondary data analysis was conducted using the Jonglei state Acute Flaccid Paralysis (AFP) surveillance case-based database from 2011 to 2020. The reason for selecting Jonglei is because it is one of the poor performing states and is chronically hit by flood and internal conflicts. Data analyses were carried out using the Microsoft Excel (2016) program, where descriptive analysis frequencies, tables, and graphs were generated.

**Results:**

the study revealed that 346 AFP cases were reported in the counties of Jonglei state from 2011 through 2020. Out of 11 counties, 11 (100%) of them have reported suspected AFP cases. Children under five years accounted for 275 (79%) of all cases. The male gender accounted for 175 (51%) of all cases. A total of 249 (72%) had received three or more doses of Oral Polio Vaccine (OPV). Non-Polio Acute Flaccid Paralysis (NPAFP) rate varies from 1.2 in 2014 to 4.4 cases per 100,000 children under 15 years in 2018. The stool adequacy ranges from 58% in 2020 to 100% in 2011.

**Conclusion:**

the performance of Jonglei´s AFP surveillance system did not meet the WHO recommended target for both major AFP surveillance indicators (non-polio AFP rate and stool adequacy) during the study period.

## Introduction

Poliomyelitis, commonly known as polio, is a disease caused by the poliovirus. It is a paralytic illness with a permanent disability. The disease primarily affects children under five years [1,2]. In May 1988, the forty-first World Health Assembly (WHA) resolved to eradicate poliovirus. The resolution gave birth to the Global Polio Eradication Initiative (GPEI). It is one of the most extensive public health interventions in the history of disease eradication. The program is mainly led by national governments in partnership with the World Health Organization (WHO), United States Centers for Disease Control and Prevention (US CDC), among others [3,4]. Since its commencement in 1988, the GPEI has made enormous progress with an over 99% reduction in global polio cases [5,6]. The GPEI has four strategies for Polio eradication: 1) strong routine immunization with the oral polio vaccine (OPV); 2) supplementary, additional doses of OPV countrywide during National Immunization Days (NIDs); 3) mop-up campaigns in the inaccessible and low immunization coverage areas; and 4) enhance Acute Flaccid Paralysis (AFP) surveillance and report. Acute Flaccid Paralysis surveillance is a key strategy for monitoring the progress of polio eradication [7]. A well-functioning AFP surveillance network offers knowledge for the preparation, execution, control, and provide evidence of eradication or absence of circulation of the disease. Acute Flaccid Paralysis surveillance remains the “gold standard” for detecting the transmission of poliovirus [7-9].

Polio eradication activities in South Sudan started in 1998 in collaborating with WHO and Organization Liberation Sudan (OLS) when the country was part of Sudan. Polio eradication activities were incorporated in the Sudan polio program until South Sudan officially moved to the African Region in October 2013 [10]. South Sudan reported the last indigenous wild poliovirus (WPV) in 2001 in Unity State, while the country was part of Sudan. In addition, the country reported two importations of WPV in 2004-2005 and 2008-2009. The 2008 -2009, outbreak resulted in a total of 64 imported cases (24 cases in 2008 and 40 cases in 2009) and spread to all states of the country except Western Bahr Ghazal. The country remained free from the wild poliovirus since June 2009 [11]. The country continues to be at risk of importation of the circulating vaccine-derived poliovirus type 2 (cVDPV2) with an outbreak of cVDPV occurring in 2014, 2015, and 2020. A total of 50 AFP cases were confirmed for the cVDPV2 with 4 from Jonglei state [12]. The study described the characteristics of reported AFP cases in the last ten years (2011 to 2020). In addition, the study evaluated surveillance performance using the WHO and nationally recommended surveillance standards and recorded lessons and challenges.

## Methods

**Study area:** Jonglei state is one of the ten states in South Sudan. It is the largest state in the country, occupying a land area of 123,070 km^2^. The estimated total population is 2.2 million according to the National Bureau of Statistics census 2008 projection. The state lies between longitudes 28 and 30° East, and latitudes 7 and 9 degrees North. Its altitude ranges between 428 and 456 meters above sea level. It comprises 11 counties, 72 Payams, and 343 Bomas.

**Selection of study area:** Jonglei is one of the former three conflict-affected states and least developed states with poor infrastructure. Access within and outside of states by road is impossible due to flooding, insecurity, and poor road structure. During the rainy season, access is possible using air transport or boats. More importantly, the state is chronically poor performing in the early detection of cases. In this regard, the author’s raised a concern and aimed to analyze the surveillance performance and compare it with WHO surveillance performance indicators and other countries.

**Study design:** a cross-section retrospective study design was used to describe the implementation of acute flaccid paralysis surveillance from 2011 to 2020.

**Study population:** the target population for this study is children below 15 years of age with sudden onset of weakness or floppiness in one or more limbs regardless of the cause and any person above 15 years old that a physician suspects polio.

**Stool sample collection and packaging:** two stool specimens of 8-10 grams (size of two adult thumbnails) were collected from AFP suspected cases within 48 hours. The specimens were stored in tightly closed containers and placed in a cold box at 2-8°C).

**Laboratory method:** two stool samples were collected from AFP cases and transported to a WHO accredited laboratory in Uganda, while the national guideline and protocol for sample transportation and testing strictly followed.

**Data collection and analysis:** we used the case-based and surveillance line-list data from 2011-2020, which was entered into MS-Excel, where all descriptive analysis frequencies, tables, and graphs were generated.

Definition of terms (the definitions are extracted from the Global Polio Eradication Initiative document)

**Suspected AFP:** “any child below 15 years of age with sudden onset of weakness or floppiness in one or more limbs regardless of the cause and any person above 15 years old that a physician suspects polio”.

**Confirmed polio case:** “a suspected case with isolation of WPV or VDPV in stool specimens collected from the suspected case or close contact”.

Non-polio AFP (NPAFP) case: discarded cases or all AFP cases excluding WPV, cVPDV2, and compatible cases.

**Non-polio AFP rate:** “number of discarded as NPAFP in children <15 years of age/number of children aged < 15 years x 100,000 per year”.

**Inadequate case:** “late stool collected >14 days from onset of paralysis or collected stool sample arrived at polio laboratory in bad condition”.

**Stool adequacy rate:** “the number of AFP cases with two stools collected ≤14 days after paralysis of onset, two stool samples collected ≥ 24-48 hours apart, and in “good condition divided by a total number of AFP cases, and multiplied by 100”.

**Compatible case:** “a suspected case with no adequate specimens and no isolation of WPV or VDPV from the case or close contacts; and residual paralysis revealed after 60 days follow up. The expert review committee classified as compatible cases due to lack of sufficient clinical and epidemiological data to rule it out polio”.

**Discarded case:** “a suspected case that was adequately investigated (including the collection of adequate stool specimens) and resulted in any of the following: no laboratory evidence of WPV or VDPV infection, inadequate specimens collected and resolution of weakness within 60 days of paralysis onset, deemed by the national expert review committee to not be compatible with poliomyelitis”.

**Ethical consideration:** ethical approval was obtained from the Research and Ethics Committee of the national Ministry of Health to use the secondary anonymized data in the analysis. Confidentiality was ensured as the line list was anonymized, and data protection measures were employed to ensure the security of the data. Administrative clearance for publication of this editorial was provided by the Ministry of Health of South Sudan and WHO (WHO e-ePub-IP-00331568-EC to publish the result. Moreover, the Research Ethics Review Board of the Ministry of Health provided clearance for the publication of the Manuscript (MoH/RERB/D.03/2022).

## Results

From 2011 to 2020 during the study period, 346 AFP cases were reported in the 11 counties of Jonglei state. The number of cases reported by year varied from 10 in 2014 to 50 in 2020. Most of the reported cases were children under five years, which accounted for 275 (79%) of all cases. Males accounted for 175 (51%) of cases. Fever at the onset of paralysis was recorded in 325 (94%) and asymmetric paralysis in 226 (65%) of all reported AFP cases (Table 1). The non-polio acute flaccid paralysis (NPAFP) attack rate in the study period ranges from 1.2 in 2014 to 4.4 cases per 100,000 children under 15 years of age in 2018. The stool adequacy varied from 58% in 2020 to 100% in 2011. Four out of eleven counties (Ayod, Nyirol, Pibor and, Pigi, did not report cases for three to five years, while Fangak and Pochalla to one to two years (Table 2). A total of 244 (71%) cases were reported more than >14 days from the onset of paralysis. Of the reported AFP cases, 312 (90%) were investigated within 48 hours of notification to the county surveillance officer. After investigation and laboratory testing, 328 (95%) of the cases were classified as discarded cases (Table 3). The immunization profile in the study period of the state indicates; 249 (72%) had three or more Oral Polio Vaccine (OPV) doses, 41 (12%) had 1 to 2 doses, 24 (7%) had zero doses (OPV0), and 32 (9%) of missing doses (Table 4). A total of 4 cVDPV2 and 14 compatible cases were also reported from different counties during the study period. Out of the total AFP suspected cases, 25% (85/246) were NP-ENT cases (4).

**Table 1:** characteristics of acute flaccid paralysis in Jonglei State, South Sudan, 2011-2020

Demographic characteristics	Parameter	2011	2012	2013	2014	2015	2016	2017	2018	2019	2020	Total
**AFP cases**	AFP cases reported	33	41	32	10	23	34	35	45	43	50	346
**Gender**	Male (N= 175, 51%)	19	18	19	6	11	18	12	23	26	23	175
	Female (N= 171, 49%)	14	23	13	4	12	16	23	22	17	27	171
**Vaccination status**	Zero doses (N=24, 7%)	0	0	0	1	0	3	0	2	6	12	24
	1-2 dose (N=41, 12%)	4	1	1	1	2	5	3	6	3	15	41
	3+ doses (249, 72%)	29	40	31	7	21	26	24	27	28	16	249
	Missing dose (32, 9%)	0	0	0	1	0	0	8	10	6	7	32
**Age**	0-5 Years (N= 275, 79%)	29	33	29	7	18	29	28	33	32	37	275
	6-10 Years (N= 43, 12%)	2	5	2	1	2	4	3	6	8	10	43
	11-15 Years (N= 12, 3%)	2	3	0	2	2	0	1	1	1	0	12
	>15 Years (N= 3, 1%)	0	0	1	0	0	0	0	1	0	1	3
	Missing years (N= 13, 4%)	0	0	0	0	1	1	3	4	2	2	13
**Clinical history**	Fever at the onset of paralysis (N= 325, 94%)	31	35	32	10	23	33	33	41	41	46	325
	Asymmetric paralysis (N= 226, 65%)	21	22	23	7	22	33	25	19	23	31	226
	Paralysis progressed within 3 days (N= 303, 88%)	29	33	25	10	19	32	33	36	39	47	303

**Table 2:** number of total AFP cases reported by county by year in Jonglei state, South Sudan 2011-2020

County/Year	2011	2012	2013	2014	2015	2016	2017	2018	2019	2020	Total
Akobo	4	2	5	1	3	5	2	5	5	4	36
Ayod	4	2	3	0	0	2	0	7	7	11	36
Bor South	6	9	4	3	9	8	11	8	4	1	63
Duk	2	3	1	1	1	6	3	2	5	4	28
Fangak	2	3	4	0	1	2	1	0	1	4	18
Nyirol	2	0	0	0	2	0	0	1	1	6	12
Pibor	1	1	0	0	0	3	5	7	3	8	28
Pigi	1	4	3	0	0	0	0	3	4	4	19
Pochalla	1	2	1	2	1	1	4	5	0	0	17
Twic East	7	9	3	2	2	4	6	4	7	4	48
Uror	3	6	8	1	4	3	3	3	6	4	41
Total	33	41	32	10	23	34	35	45	43	50	346

**Table 3:** main surveillance indicators for acute flaccid paralysis by County and by year in Jonglei State, South Sudan, 2011-2020

Parameter	Main indicators	2011	2012	2013	2014	2015	2016	2017	2018	2019	2020
Akobo	NPAFP rate	3.7	1.8	4.5	1.1	3.3	5.4	2.1	5.1	4.9	3.8
	Stool adequacy (%)	100	100	80	100	100	60	100	60	40	50
Ayod	NPAFP rate	4.1	2	3	0	0	2.1	0	7.1	6.9	5.7
	Stool adequacy (%)	100	100	100	0	0	100	0	100	71	64
Bor South	NPAFP rate	3.5	5.1	2.3	7.2	21.1	18.2	24.3	17.2	2.4	0.6
	Stool adequacy (%)	100	89	75	100	100	100	91	100	100	100
Duk	NPAFP rate	6.3	9.1	3	1.4	1.4	8.2	4	2.6	8.3	8.1
	Stool adequacy (%)	100	67	100	100	100	100	100	100	80	100
Fangak	NPAFP rate	2.3	3.4	4.5	0	1.3	2.6	1.3	0	1.2	2.3
	Stool adequacy (%)	100	100	100	0	100	100	100	0	100	50
Nyirol	NPAFP rate	2	0	0	0	2.1	0	0	0.9	0	6.1
	Stool adequacy (%)	100	0	0	0	50	0	0	0	0	33
Pibor	NPAFP rate	0.8	0.8	0	0	0	6.8	11	12.8	1.8	4.5
	Stool adequacy (%)	100	100	0	0	0	67	60	86	33	13
Pigi	NPAFP Rate	3	11.7	8.8	0	0	0	0	2.7	4	5.2
	Stool adequacy (%)	100	100	100	0	0	0	0	33	5	7
Pochalla	NPAFP rate	4.1	7.9	3.9	1.4	0.7	0.7	1.9	3.1	0	0
	Stool adequacy (%)	100	100	100	100	100	100	75	100	0	0
Twic East	NPAFP rate	17.5	21.8	7.3	3.7	3.6	7	10.1	6.6	11.1	6.2
	Stool adequacy (%)	100	89	100	50	100	100	100	75	86	100
Uror	NPAFP rate	2.9	5.7	7.5	0.9	3.4	2.5	2.4	2.4	4.6	3
	Stool adequacy (%)	100	100	100	100	100	67	100	100	67	75
Jonglei State	NPAFP rate	3.6	4.3	3.4	1.2	2.6	3.7	3.6	4.4	3.9	3.8
	Stool adequacy (%)	100	93	94	90	96	88	89	84	67	58

**Table 4:** classification of acute flaccid paralysis in Jonglei State, South Sudan, 2011-2020

Parameter	2011	2012	2013	2014	2015	2016	2017	2018	2019	2020	Total
AFP cases reported	33	41	32	10	23	34	35	45	43	50	346
% of notification with in <7 days of onset	79	78	72	70	74	88	77	60	70	50	71
% of investigation with in <2 days from notification	100	100	97	100	100	97	80	87	88	72	90
# of inadequate cases	0	2	2	1	1	4	4	7	10	21	52
% of Npent cases isolated	27	29	25	30	35	29	26	16	19	22	25
Number of WPV cases	0	0	0	0	0	0	0	0	0	0	0
Number of cVDPV2 cases	0	0	0	0	0	0	0	0	0	4	4
Number of compatible cases	0	0	0	0	0	0	1	2	4	7	14
Number of discarded cases	33	41	32	10	23	34	34	43	39	39	328

## Discussion

The study showed that, although AFP cases were reported from all age groups in the state, the majority (79%) of them were children below the age of five years, this finding is consistent with other states within the country and similar studies conducted in Kenya, Ethiopia, Somalia, and Nigeria [1,3,13,14]. Besides, our findings of gender proportionality of reported AFP cases were consistent with studies conducted in Kenya, Ethiopia, Somalia, and Nigeria with proportions of 55%, 58%, 60%, and 56% respectively [1,3,13,14]. However, no disparity was found while compared with other states within the country. The polio vaccination status of the reported cases was also far from adequate to ensure the establishment of herd immunity in the population. The immunization status of reported cases in Jonglei state is lower than the national figure reported during the same period (66% in 2011 and 50% in 2019). The OPV 3 coverage (percentage of surviving infants who received the 3rd dose of polio-containing vaccine). Similar studies conducted in Kenya from 2016-2018 and in Ethiopia from 2005 to 2015 on reported AFP cases also revealed the immunity profile of AFP cases reported was higher than Jonglei state [1,3]. This finding is also supported by the national immunization coverage survey finding in 2017 that most conflict-affected states including Jonglei have documented a very low level of immunization coverage compared to none-conflict affected states [15,16]. Moreover, perennial flooding had played a critical role in low immunization coverage and led to children´s exposure to cVDPV2 [17]. On the other hand, the proportion of cases with zero doses and those whose immunization status was not known or missing was higher than the national figure for the same period [15]. Most of the counties in the state have reported at least one AFP and NP-AFP case. The overall NP_AFP rate was two cases per 100,000 in the under 15 years population. Though, in 2014, 45% (5/11) of the counties were silent, which made the state fail to meet the NP-AFP target for the year. The possible reason for the decrease in AFP case reporting and being silent in 2014 was as a result of the conflict in 2013 conflict, which impacted the surveillance system of the state even after the conflict [15]. The trend analysis of the NP-AFP rate was improving from 2015 to 2020, while the stool adequacy rate revealed a decreasing trend with minor fluctuation during the same period [15].

The stool adequacy across all counties chronically low compared to other states within the country. In the same period, even states with a similar situation (unity and upper Nile) achieved the target. One of the factors that may have contributed may be the ongoing inter-clan conflict than any other states on top of the consequence of previous war [18]. The inconsistent and low stool adequacy rate in the state during 2019 and 2020 was due to the occurrence of massive flooding in most of the counties. The situation was aggravated by the untoward consequences of the COVID-19 pandemic in the country. This assumption was also supported by a study conducted globally. There was a 33% decline in AFP case reports and compromised stool adequacy between 2019 and 2020 due to the COVID-19 pandemic [19,20]. Most of the counties in the state have not attained either one of the two major surveillance performance indicators minimum target. Twic East county persistently attended NP-AFP target below two cases per 100,000 children aged < 15 years per year throughout the study period. The proportion of AFP cases notified within seven days of the onset of paralysis was below 80% during the study period, except in 2016 [21,22]. On the other hand, the proportion of AFP cases investigated within 48 hours from the date of notification was above 80% except in 2020. As for the “percentage of cases investigated within 48 hours of notification”, the finding is consistent with national figures and most countries in Africa [23]. Close to 15% (52/346) of AFP cases were classified as inadequate and for which, most of these cases were reported late. This signifies that the sensitivity of surveillance network and early detection of AFP cases in the state and counties were suboptimal which doubt the system for undetected circulation virus. The situation is compounded by poor and fragile health systems, insecurity, and natural disaster such as flooding [24]. The none polio AFP NP-AFP) rate for the state was, 1.2 in 2014 to 4.4 per 100,000 children under 15 years in 2018. This finding is comparable with the most recent findings of the neighboring countries such as Kenya and Ethiopia with an average rate of 1.3% and 7.9% respectively [1,3]. However, through the period of analysis, no county attend both indicators consistently, while in other states in most of the counties consistently attained and maintained throughout the study period (polio-free documentation).

## Conclusion

The performance of Jonglei´s AFP surveillance system did not meet the WHO recommended minimum target or threshold for both major AFP surveillance indicators (non-polio AFP rate and stool adequacy) during the period studied. Performances of four counties remain a concern and were negatively impacting the performance of the state in attaining the indicators irrespective of external factors and various limitations. Early detection and reporting of cases were sub-optimal. Though the number of AFP cases reported increases slightly year after year, the stool adequacy report showed a decreasing trend even in the counties where community-based surveillance workers (CBS) are deployed. The immunity status of reported cases was also significantly low. To strengthen Jonglei state´s polio-free status, all stakeholders including health facility surveillance focal persons, WHO field supervisors, WHO field assistants, CBS workers need to have an in-depth awareness to improve the sensitivity of AFP surveillance across all counties in the state. In addition, strengthening partners’ coordination will improve routine immunization coverage and ensure all target children have received the required doses at the right age.

### What is known about this topic

- South Sudan and the African continent were certified of circulating wild poliovirus-free status in August 2020;
- Acute flaccid paralysis surveillance is one of the strategies for the polio eradication;
- Surveillance performance indicators are being monitored at the state and counties level to achieve polio eradication.

### What this study adds

- The study being the first surveillance paper in the state provided, it had compared relevant information on surveillance performance over years with national, regional, and global standards;
- The study also identified challenges and recommends ways of improving the surveillance performance indicators in the state.
